# Effects of Ultra-High Pressure Synergistic Enzymatic Hydrolysis on Flavor of *Stropharia rugoso-annulata*

**DOI:** 10.3390/foods12040848

**Published:** 2023-02-16

**Authors:** Chenligen Bao, Minghang Xin, Keyu Su, Chunbo Guan, Dawei Wang

**Affiliations:** School of Food Science and Engineering, Jilin Agricultural University, Changchun 130118, China

**Keywords:** *Stropharia rugoso-annulata*, ultra-high pressure, enzymatic hydrolysates, electronic nose, electronic tongue, flavor

## Abstract

In this study, using gas chromatography-mass spectrometry (HS-SPME-GC-MS), electronic nose (E-nose), high performance liquid chromatography (HPLC), and electronic tongue (E-tongue) to analyze the effect of ultra-high pressure (UHP) synergistic enzymatic hydrolysis on the flavor compounds of enzymatic hydrolysates of *S. rugoso-annulata*. The results demonstrated that 38 volatile flavor substances were identified in the enzymatic hydrolysates of *S. rugoso-annulata* treated at atmospheric pressure and 100, 200, 300, 400, and 500 MPa, mainly 6 esters, 4 aldehydes, 10 alcohols, 5 acids, and 13 other volatile flavor substances, and the most kinds of flavor substances reached 32 kinds when the pressure was 400 MPa. E-nose can effectively distinguish the overall changes of enzymatic hydrolysates of *S. rugoso-annulata* treated with atmospheric pressure and different pressures. There was 1.09 times more umami amino acids in the enzymatic hydrolysates at 400 MPa than in the atmospheric pressure enzymatic hydrolysates and 1.11 times more sweet amino acids at 500 MPa than in the atmospheric pressure enzymatic hydrolysates. The results of the E-tongue indicate that the UHP treatment increased umami and sweetness and reduced bitterness, which was also confirmed by the results of amino acid and 5′-nucleotide analysis. In conclusion, the UHP synergistic enzymatic hydrolysis can effectively improve the overall flavor of the enzymatic hydrolysates of *S. rugoso-annulata*; this study also lays the theoretical foundation for the deep processing and comprehensive utilization of *S. rugoso-annulata*.

## 1. Introduction

*Stropharia rugoso-annulata* (*S. rugoso-annulata*) is a precious edible fungi of the genus stropharia recommended by the United Nations Food and Agriculture Organization (FAO) in developing countries, which has become one of the top ten mushrooms in the international mushroom market [1]. It has been reported that *S. rugoso-annulata* is a good source of protein, polysaccharides, minerals, and vitamins, and it is a high-quality nutritional health food [2,3]. The recommended *S. rugoso-annulata* shows potential antioxidant, antiviral, antitumor, and hypoglycemic due to various bioactive components, which have nutritional and medicinal value [4,5]. *S. rugoso-annulata* are highly favored by consumers. At present, most of the edible mushrooms are supplied to the market in dried form [6], and their flavor is bland. If the flavor enhancement process can be carried out, the application and consumption of the mushroom can be greatly increased, and the development of the mushroom industry can be effectively promoted.

Ultra-high pressure (UHP) belongs to non-thermal processing technology that uses liquid as a pressure transmission medium to apply more than 100 MPa pressure to the treated material to improve the performance of the treated material or kill harmful microorganisms, also known as static high static pressure technology [7], which is one of the hottest food processing technologies in the international arena. In recent years, UHP technology has been widely used in the extraction and processing of natural macromolecular components of plants and flavor improvement of foods, etc. It has been reported that the total sugar and galacturonic acid content of UHP-treated large leaf yellow tea increased; UHP treatment could significantly change the primary structure and surface morphology of large leaf yellow tea polysaccharides [8]. According to the research work, combined UHP and carnosine treatment could effectively inhibit the fishy off-odor volatile substances in snakehead and better preserve the color and texture of the fillets [9]. In another study, the content of characteristic volatile compounds in vacuum freeze-dried strawberry slices pretreated with UHP significantly increased, and the taste of strawberry slices improved [10].

Enzymatic hydrolysis can fully release the flavor and nutrients in food; however, traditional enzymatic hydrolysis has the defects of long enzymatic time, high energy consumption of enzymatic hydrolysis, low enzyme utilization, low substrate conversion rate, and low enzymatic efficiency [11], etc. Some studies have shown that appropriate pretreatment before enzymatic hydrolysis usually changes the type and quantity of enzymatic hydrolysates then improves the comprehensive quality of enzymatic hydrolysates; thus, a combination of new technology with enzymatic hydrolysis becomes an important method. For example, using enzymatic hydrolysis extraction after single ultra-high pressure pre-treatment could effectively improve fish oil yield and reduce nutritional losses [12]. Ultrasound pretreatment increased the content of umami amino acids, sweet amino acids, nucleotides, and succinic acid in the enzymatic hydrolysis of cod head and the type and relative content of volatile compounds increased with ultrasound power [13]. Rice pretreated with a pulsed electric field [14] showed an increase in protein hydrolysis; a decrease in the content of unpleasant volatile flavor substances, such as benzaldehyde; and an increase in the content of volatile flavor substances with fruity and sweet aromas, such as acetophenone.

Therefore, new pretreatment techniques can be used as structural modifications before enzymatic hydrolysis to increase the degree of enzymatic hydrolysis and improve the functional properties of the enzymatic hydrolysates. Flavourzyme^®^ are a mixture of endo and exo peptidases that accelerate the hydrolysis of proteins, which belongs to the class of non-specific proteases. It breaks down proteins into small molecule peptides and free amino acids, which helps to improve the flavor and nutritional value of foods. In this study, we used Flavourzyme^®^ and investigated the effect of ultra-high pressure synergistic enzymatic hydrolysis on the flavor substances of the enzymatic hydrolysates of *S. rugoso-annulata*. The techniques like gas chromatography-mass spectrometry (HS-SPME-GC-MS), electronic-nose (E-nose), high performance liquid chromatography (HPLC), and electronic-tongue (E-tongue) were used to analyze the flavor substances of the enzymatic hydrolysates of *S. rugoso-annulata* to determine the conditions of ultra-high pressure synergistic enzymatic hydrolysis that can improve the flavor of the enzymatic hydrolysates of *S. rugoso-annulata* and to lay the theoretical foundation for the deep processing and comprehensive utilization of *S. rugoso-annulata*.

## 2. Materials and Methods

### 2.1. Materials

The fresh *S. rugoso-annulata* was provided by Xinxin Vegetable Planting Cooperative (Meihe, Jilin, China). 2-Methyl-3-heptanone was purchased from Maclean Biochemical Technology Co., Ltd. (Shanghai, China). The *n*-alkanes (C_8_~C_20_) were purchased from Sigma-Aldrich Co., Ltd. (Shanghai, China). The 5′-nucleotides standards obtained from Sigma-Aldrich (St. Louis, MO, USA). The amino acids mixture standard solution was purchased from Waters (Shanghai, China). Flavourzyme^®^ was purchased from Solaibao Technology Co. (Beijing, China).

### 2.2. Sample Preparation

The cleaned *S. rugoso-annulata* was dried at 40 °C to the moisture content ≤6% and then crushed into a powder with a particle size of 0.125 mm, weighed 6 portions of *S. rugoso-annulata* powder (100 g each), configured into a mixture according to ratio of *S. rugoso-annulata* powder to water is 1:15 (*m*/*v*) (i.e., 1 g sample and 15 mL water), packed into sterile bags and vacuum-packed, and Flavourzyme^®^ was added after processing at atmospheric pressure 100, 200, 300, 400, and 500 MPa for 15 min, respectively. The amount of Flavourzyme^®^ added was 3000 U/g, pH was adjusted to 7.0 with 0.01 mol/L NaOH solution after 2 h enzymatic hydrolysis at 40 °C, and the hydrolysates were heated at 100 °C maintained for 5 min to inactivate the enzyme. The samples were obtained by vacuum freeze-drying the enzymatic hydrolysates of *S. rugoso-annulata*. The sample of atmospheric pressure enzymatic hydrolysates was used as the control sample.

### 2.3. Determination of Volatile Flavor Compounds by HS-SPME-GC-MS

Volatile compounds were detected by HS-SPME-GC-MS (TSQ 9000, Thermo Fisher, Shanghai, China) according to Hou’s [15] method with modifications. Add 0.8 g of samples to 8 mL flat bottom top empty vial. An amount of 1 μL of 2-methyl-3-heptanone (0.0816 μg/μL) was added to the samples as the internal standard (IS); the vial was sealed with a cap with a polyvinyl chloride spacer, heated at 50 °C for 20 min to equilibrium, was inserted with 50/30 µm DVB/CAR/PDMS (divinyl benzene/carboxy/polydimethylsiloxane) fiber needle into the flat bottom top empty vial for 30 min to extract volatile compounds, then had the fiber needle removed and inserted into the sample inlet of the GC-MS instrument and was analyzed at 250 °C for 2 min.

GC: Chromatographic column was HP-INNO Wax (30 m × 0.25 mm × 0.25 μm, Agilent J&W, Santa Clara, CA, USA), the start temperature of column was set to 40 °C and kept for 5 min; the temperature was increased to 150 °C, maintained for 1 min at a rate of 5 °C/min, increased to 220 °C, held for 2 min at a rate of 8 °C/min, then increased to 230 °C for 2 min at a rate retained for 10 °C/min. The inlet temperature was 250 °C. Helium (99.999%) was used as the carrier gas at the rate of 1.0 mL/min, and the split ratio was 10:1.

MS: The energy of electron impact ionization was set to 70 eV; the temperature of transfer line and ion source was set to 230 °C and 240 °C, respectively. Solvent delay was set at 2.0 s, full scan mode was set, and the mass scan range was selected from 33 *m*/*z* to 550 *m*/*z*.

Qualitative and quantitative analysis [16]: the volatile compounds were performed according to comparing mass spectra with the National Institute of Standards and Technology (NIST) and supported by retention indexes. The retention indexes (RI) were calculated with running *n*-alkanes (C_8_~C_20_) under the same chromatographic conditions. RI of target analytes was calculated according to Equation (1). The content of volatile compounds was calculated using 2-methyl-3-heptanone as the IS and following the Equation (2).

(1) $$RI=100\times \left[n\times \frac{{RT}_{m}-{RT}_{n}}{RT_{n+1}-{RT}_{n}}\right]$$

where $RT_{m}$ is the corrected retention time of component to be detected, *n* is the number of carbon atoms of the previous *n*-alkane flowing out of the unknown, and *n* + 1 is the number of carbon atoms of the next *n*-alkane. $RT_{n+1}$, $RT_{n}$ are the retention times of *n*-alkanes having *n* and *n* + 1 carbon atoms, and the magnitude relationship between them is *n* < *m* < *n* + 1.

(2) $${The\;content\;of\;volatile\;compounds}_{\left(\mu g/g\right)}=\frac{C_{is}\times V_{is}\times S_{c}}{M_{s}\times S_{is}}$$

where $C_{is}$ (μg/μL) is the concentration of IS, $V_{is}$ (μL) is the volume of internal standard, $S_{c}$ is the chromatographic peak area of the volatile compound, $S_{is}$ is the chromatographic peak area of IS, and $M_{s}$ (g) is the quality of sample.

### 2.4. Determination of E-Nose

The E-nose is a device with the ability to identify single and complex gases consisting of multiple gas-sensitive sensors with overlapping properties with each other and an appropriate pattern classification method. The PEN3 E-nose (PEN 3 Airsence, Schwerin, Germany) is composed of 10 heated metal oxide sensors array, gas flow control system, and analysis and control software. The metal oxide semiconductors have different selectivity and sensitivity to volatile compounds with a certain degree of specificity. The 10 gas sensors of the E-nose are described as shown in Table 1. An amount of 0.05 g of the sample was weighted, put in the vial, and covered with the cap with PTFE gas-tight spacer. The volatile compounds were balanced for 30 min at room temperature, and the gas was aspirated at the top of the vial with the E-nose probe and analyzed to determine its volatile flavor substance. The parameters were set by the E-nose: testing time was 60 s and flushing time was 90 s, internal flow rate was 300 mL/min, injection flow rate was 300 mL/min, and each group conducted 3 parallel experiments.

### 2.5. Determination of E-Tongue

In this study, we used the SA402B taste sensing system (Intelligent Sensor-tech Co., Ltd., Atsugi, Japan), which has an artificial lipid membrane sensor that simulates the taste sensing system of living organisms and can detect changes in membrane potential resulting from electrostatic or hydrophobic interactions between various taste substances and artificial lipid membranes. After weighing 1 g of sample, it was dissolved with 50 mL(*w*/*v*) deionized water, heated at 50 °C for 10 min, and centrifuged at 3800 rpm for 20 min. The supernatant was taken and fixed to 100 mL with ultrapure water for the assay.

### 2.6. Determination of Amino Acids

Amino acid content in the samples was measured using an amino acid analyzer (L-8900, Hitachi Co., Tokyo, Japan). An amount of 1.0 g of sample was weighed accurately, sulfosalicylic acid (3 mL, 10 g/L) and EDTA (1.5 mL, 10 g/L) were added, and the extract was extracted with ultrasonication for 1.5 h. After 12 h at 4 °C, the extract was dissolved again with 6 mol/L hydrochloric acid, fixed to 10 mL, and passed through a 0.45 μm microporous membrane for the analysis.

### 2.7. Determination of 5′-Nucleotides

The 5′-nucleotide assay was performed using high performance liquid chromatography (U3000, Thermo, Shanghai, China) with reference to the method in the literature [17]; 0.5 g of the sample was added to 30 mL of distilled water, and the extraction was heated at 50 °C for 1 min. After centrifuging at 3800 rpm for 20 min, the above operation was repeated twice for the residue, combining all supernatants, fixing the volume of distilled water to 10 mL, and filtered through a 0.45 μm filter membrane for detection.

Chromatographic column: ACQUITY UPLC BEH C18 (2.1 mm × 100 mm, 1.7 μm) was used. The column temperature was 30 °C, UV detector wavelength was 260 nm, and sample injection volume 10 μL. The mobile phase consisted of 0.02 mol/L dipotassium hydrogen phosphate (A) and acetonitrile (B) (99.5:0.5, *v*/*v*). The flow rate was 1.2 mL/min.

### 2.8. Statistical Analysis

All experiments were repeated in triplicate. Tbtools software was used for heatmap analysis of volatile flavor substances, Origin 2021 (OriginLab, Northampton, MA, USA) software was used for principal component (PCA), and radar plot analysis of the data and the unscrambler was used for PLS analysis. IBM SPSS Statistic 19 was used to analyze significance of difference.

## 3. Results

### 3.1. Analysis of Volatile Flavor Compounds

**Figure 1:**
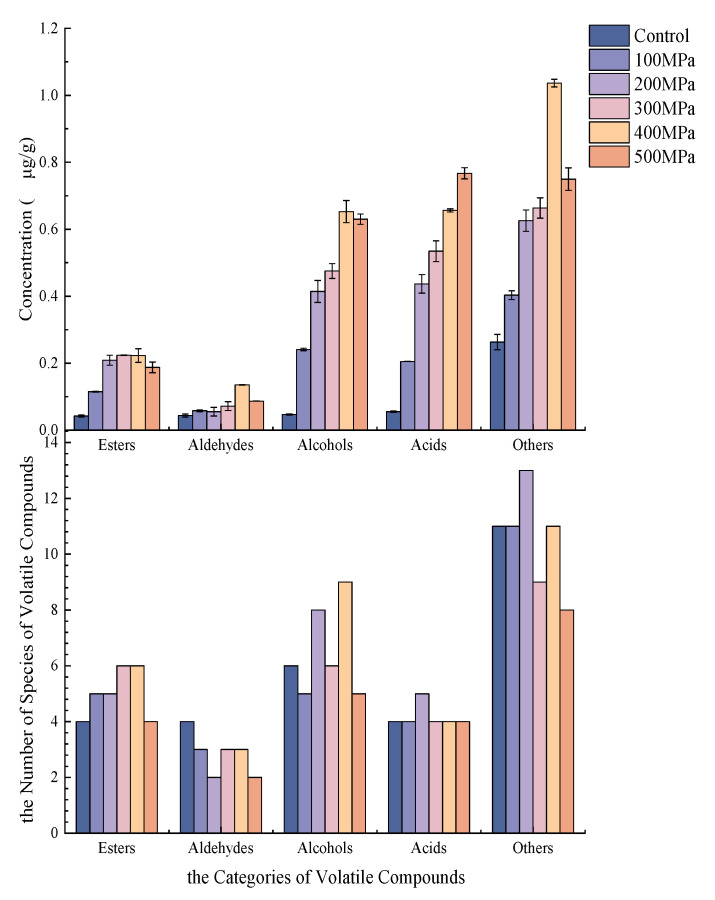
The number of species and concentration of volatile composition in enzymatic hydrolysates of *S. rugoso-annulata* after different ultra high pressure.

As shown in Table 2, a total of 38 volatile flavor substances were identified in the enzymatic hydrolysates of *S. rugoso-annulata* treated at atmospheric pressure and 100, 200, 300, 400, and 500 MPa, which included 6 esters, 4 aldehydes, 10 alcohols, 5 acids, and 13 other types of volatile flavor substances, and with the change of pressure, the composition and content of volatile flavor substances were significantly different in the enzymatic hydrolysates of *S. rugoso-annulata*, which can be seen in Figure 1. An amount of 28, 27, 31, 27, 32, and 22 volatile compounds were detected in the control sample at 100 MPa, 200 MPa, 300 MPa, 400 MPa, and 500 MPa, respectively.

Esters can be obtained with esterification reactions, i.e., the reaction of organic acids and alcohols [18], generating esters along with a molecule of water, in which acyclic hydroxycarboxylic acids can form lactones by intramolecular esterification. Compared with the control sample, the UHP treatment increased the type and content of esters and produced two new types of substances, ethyl acetate and vinyl acetate, which add a sweet aroma and pineapple aroma to the sample [19]. In addition, the relatively high content of hexyl formate, γ-valerolactone, and gamma-butyrolactone at 400 MPa contribute meat and coconut aromas to enzymatic hydrolysates of *S. rugoso-annulata*.

Aldehydes are volatile flavor substances that are abundant in edible mushrooms and also have relatively low odor thresholds [20], which usually brings special aroma to foods. Ultra-high pressure treatment reduced the content of 3-butanolal. However, the total content of aldehydes showed a trend of first increasing and then decreasing with the increase in ultra-high pressure, reaching a maximum content of 0.1355 μg/g at 400 MPa, while valeraldehyde, hexanal, and nonanal were the most abundant, providing fruity aromas, citrus aromas, and fatty aromas to the enzymatic hydrolysates of *S. rugoso-annulata* [21], and hexanal is responsible for green aromatic taste [22].

Alcohols, whose precursors are mainly polyunsaturated fatty acids, are generally a class of volatile flavor substances with a high odor threshold resulting from lipid oxidation [23]. After the UHP treatment, the content of alcohols in the enzymatic hydrolysis tended to increase and then decrease, with the highest content reaching 0.6527 μg/g at 400 MPa. The UHP treatment generated four alcohols, namely ethanol, 1-octanol,2-butyl, 2-heptanol, and propanediol; in addition, the content of ethanol, 2,3-butanediol, linalool, and phenyl ethanol increased with increasing ultra-high pressure. Ethanol imparted fruit, floral, and wine aromas to the enzymatic hydrolysates of *S. rugoso-annulata*, and phenylethanol imparted fruit and rose aromas [24].

Other substances produced during the ultra-high pressure synergistic enzymatic hydrolysis treatment of *S. rugoso-annulata* also contribute to the formation of flavor. Alkanes possess a relatively high odor threshold [25], resulting in a low impact to the volatile flavor of the enzymatic hydrolysates of *S. rugoso-annulata*. Pyridines have a low threshold and have fatty, roasted, and meaty aromas [26] and play an important role in improving the flavor of the enzymatic hydrolysates of *S. rugoso-annulata*. Pyrazines have a pleasant aroma, are important flavoring agents for fermented and baked foods, and have a significant synergistic effect with other substances [27]. The ultra-high pressure treatment increased the content of 2.6-dimethylpyrazine up to 0.0464 μg/g at 400 MPa, which played a role in flavor enhancement.

**Figure 2:**
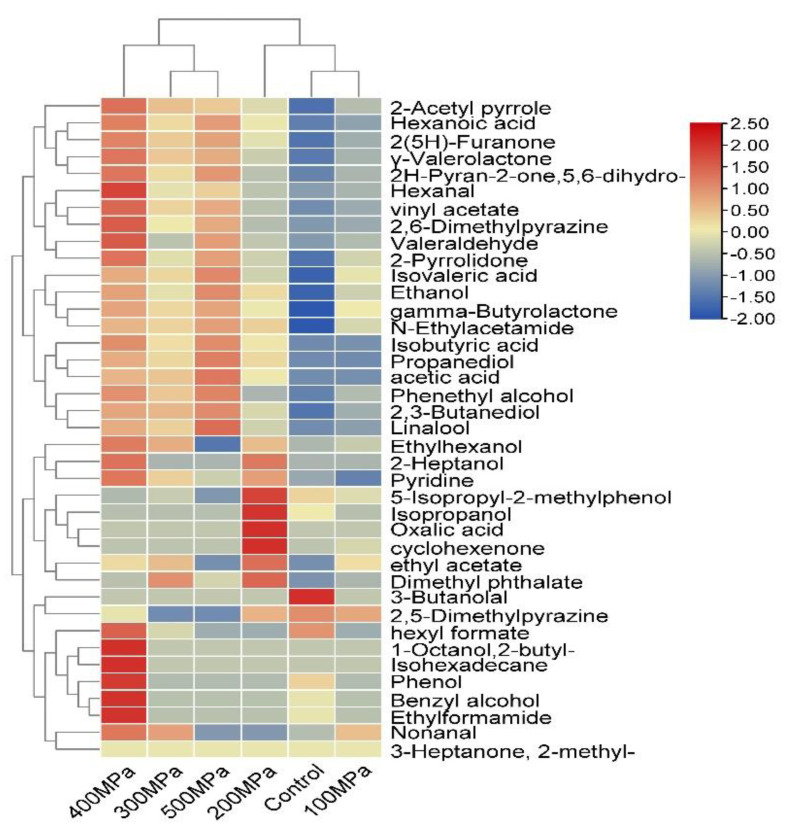
Cluster heat map of volatile flavor compounds of enzymatic hydrolysates of *S. rugoso-annulata* treated with different ultra high pressure.

To further analyze the volatile flavor substances, the volatile flavor substances of the six samples were plotted as a thermogram as shown in Figure 2, and it can be visually seen that the volatile flavor substances of the six samples have obvious differences. Blue represents low content, red represents high content, and the darker color in the heat map represents the higher content of the ingredient. It can be seen from the figure that 18 common substances, such as propyl pentolactone, 4-hydroxybutyrate lactone, valeraldehyde and 2(5H)furanone, were detected in the six samples; compared with the control group, the ultra-high pressure synergistic enzymatic hydrolysis treatment generated 10 flavor substances. The pressure of 400 MPa resulted in the largest variety of volatile flavor substances, reaching 32, mainly in the form of coconut aroma, rose aroma, nut aroma, and fat aroma, while the 100 MPa and 200 MPa samples had similar volatile flavor substances, and the volatile flavor markers were clustered into one group in the 300 MPa, 400 MPa, and 500 MPa samples.

In summary, the flavor of the UHP synergistic enzymatic hydrolysis of *S. rugoso-annulata* is produced by the interaction and coordination of many volatile compounds and acting on the human olfactory organs rather than being expressed by a single compound.

### 3.2. E-Nose Data Analysis

**Figure 3:**
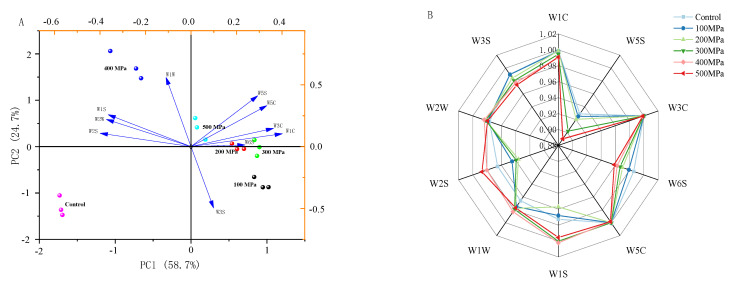
Principal component analysis (**A**) and radar Chart (**B**) of the E-nose data of enzymatic hydrolysates of *S. rugoso-annulata* treated with different ultra high pressure.

The E-nose is sensitive to volatile flavor substances of samples [28]. In this study, the overall flavor of samples was examined using the E-nose equipped with 10 types of sensors. A principal components analysis (PCA) analysis was carried out on the volatile flavor substances of the enzymatic hydrolysates of S. rugoso-annulata. The results summarize the overall relationship between the attributes and the samples. As shown in the Figure 3A, it can be seen that the contribution rate of PC1 was 58.7% and that of PC2 was 24.7%, indicating that PC1 and PC2 could reflect the overall information of the samples well. The relatively good degree of data point aggregation of the same sample in the figure indicates that the repeatability and stability of the same sample are relatively good; the control group is obviously distributed differently from other sample areas, and the dispersion degree among samples was good. W1W is associated with the sample of 400 MPa, W6S is associated with samples of 200 MPa and 300 MPa and W5S, and W5C is associated with sample of 500 MPa. Thus, there were significant differences in the volatile flavor substances of the six samples, and the E-nose could accurately and effectively differentiate them.

Small changes in volatile flavor substances led to differences in the response values of the E-nose sensors. As shown in Figure 3B, the response values of W5S, W6S, W1S, W1W, W2S, and W3S sensors were significantly different; this showed that these five sensors could distinguish samples treated with different pressures, and the ultra-high pressure treatment increased the content of pyrazines, ethanols, and aromatic, sulfur-containing substances in the enzymatic hydrolysates of *S. rugoso-annulata*, which was consistent with the results of the HS-SPME-GC-MS analysis; in contrast, the response values were small for samples at sensor W1C, W3C, W5C, and W2W, but the signal intensities were still different.

### 3.3. Correlation Analysis between E-Nose and GC-MS

**Figure 4:**
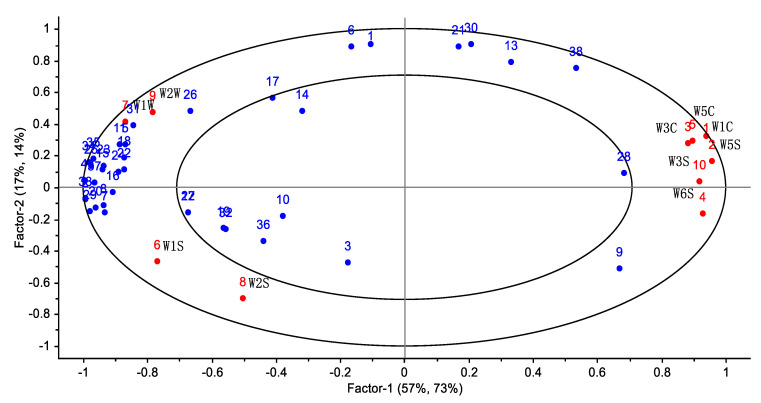
PLS plot showing the correlation between volatile components and E-nose response value.

The content of 38 volatile compounds (blue spot in Figure 4) and the response values of the E-nose sensors (red spot in Figure 4) were used as the object (Volatile compound numbering is consistent with Table 2), the PLS method was used to analyze the correlation between the content of the volatile compounds as the independent variable, and the response values of the 10 sensors were used as the dependent variables, as shown in the Figure 4. The large and small ellipses indicate 50% and 100% of the variance explained, respectively, and the dependent variables are in this range, indicating that the model contains information that can explain the correlation between the volatile compounds and the sensors. The shorter distance between the sensor and each volatile compound represents the higher correlation. Volatile flavor substances, such as n-ethylacetamide, ethanol, and gamma-butyrolactone, correlated well with W1W. Pyridine correlated closely with W2W. Phenol, benzyl alcohol, and ethylformamide were closely linked to the W2S. 1-Octanol,2-butyl-, isohexadecane, benzyl alcohol, and ethylformamide were relatively close to the W1S. These substances are positively correlated with the corresponding E-nose sensors. The above results indicate that volatile compounds have good correlation with the E-nose sensor.

### 3.4. E-Tongue Data Analysis

The results of this study showed that the sourness and astringency flavors of the samples were lower than the tasteless values, so this paper analyzed bitterness, aftertaste-B, umami, richness, saltiness, and sweetness.

We observed through Table 3 that the umami value of the samples gradually increased with the increase of ultra-high pressure; the umami of 400 MPa had the highest value, which may be because the UHP synergistic enzymatic hydrolysis treatment made the samples release more umami amino acids. The bitterness value showed a trend of increasing then decreasing with the increase of ultra-high pressure, and the bitterness value was the lowest when the pressure was 400 MPa. The protease hydrolysis of proteins into peptides and amino acids occurred, and the short peptides that were not involved in the chemical reaction contribute to the formation of salty taste [29]. With the increase in pressure, the short peptides decreased, which may be the reason for the decrease in saltness value in the samples. The above indicates that the UHP treatment helps to reduce the bitterness in the enzymatic hydrolysates of *S. rugoso-annulata* and can increase the umami and sweetness.

### 3.5. Amino Acids Analysis

The characteristic strong umami in edible mushrooms is mainly derived from their richness in substances like amino acids and taste-presenting nucleotides, which are the main products of protein hydrolysis [30]. In this study, 17 amino acids were detected in six samples (Table 4), and the amino acids were classified according to taste characteristics [31]: umami amino acids, sweet amino acids, bitter amino acids, and tasteless amino acids. Asp and Glu are not only typical umami flavor substances in edible mushrooms, but they are also precursors of many volatile flavor compounds and have good synergistic effects with taste-presenting nucleotides [32]. When the pressure was 400 MPa, Asp and Glu reached the highest values, which were 2.59 g/100 g and 5.89 g/100 g, respectively. Sweet amino acids included Thr, Ser, Gly, Pro, and Ala, with the highest total sweet amino acids content at 500 MPa compared with the control sample, reaching 9.14 g/100 g. Phe, His, Val, Met, Ile, Leu, and Arg are representatives of bitter amino acids, which decreased the most at 400 MPa compared with the control sample, with a decrease of 75.36%, probably because the UHP treatment unfolded the structure of the proteins, exposing more cleavage sites and improving the enzymatic effect, and according to the study, deep enzymatic digestion can reduce the intensity of bitterness [33]. In conclusion, UHP synergistic enzymatic hydrolysis can improve the overall flavor of the enzymatic hydrolysates of *S. rugoso-annulata*; when the pressure was 400 MPa, the enzymatic hydrolysates of *S. rugoso-annulata* were richer in taste active substances.

### 3.6. 5′-Nucleotides Analysis

Nucleotides are mainly produced by the degradation of nucleic acids under specific biological enzymes, which are an important part of non-volatile flavor substances, among which 5′-GMP, 5′-IMP, 5′-UMP, 5′-CMP, and 5′-AMP are abundant in edible mushrooms and have synergistic effects with amino acids [34]. 5′-AMP was not detected in the enzymatic hydrolysates of *S. rugoso-annulata* after ultra-high pressure synergistic enzymatic hydrolysis treatment, probably due to species differences [35]. On the other hand, it may be because the 5′-AMP is degraded to ribose during the drying process. As shown in Table 5, after UHP treatment, the nucleotide contents in the enzymatic hydrolysates of *S. rugoso-annulata* became significantly higher than those in the control sample. The enzymatic hydrolysates of *S. rugoso-annulata* contained relatively high levels of 5′-CMP, with the highest level reaching 400 MPa (414.409 mg/100 g), an increase of 2.656 mg/100 g, 5′-GMP, 5′-IMP, and 5′-UMP played a strong role in taste presentation, in which 5′-IMP collaborated with sweet amino acids to enhance unami [36], and the enzymatic hydrolysates of *S. rugoso-annulata* treated with ultra-high pressure contained 5′-GMP, 5′-IMP, and 5′-UMP reached the highest levels of 400 MPa (5.364 mg/100 g), 400 MPa (43.098 mg/100 g), and 200 MPa (23.579 mg/100 g), respectively. 5′-GMP and 5′-IMP had a potentiating effect on meat flavor and sweetness, respectively, and were much stronger than monosodium glutamate alone [37].

## 4. Conclusions

In this paper, we studied the effect of ultra-high pressure synergistic enzymatic hydrolysis on the flavor of the enzymatic hydrolysates of *S. rugoso-annulata*. A total of 38 volatile flavor compounds were detected and identified, including esters, aldehydes, alcohols, and other substances, and the most types of volatile flavor substances were generated when the pressure was 400 MPa, reaching 32, which improved the flavor richness of the enzymatic hydrolysates, increased the overall flavor of this sample, and increased the fruity, rose, nut, and cocoa aromas. The differences in the volatile flavor substances among the samples were visualized by plotting the clustering heat map. E-nose could clearly distinguish the odor profiles of the enzymatic hydrolysates of *S. rugoso-annulata* treated with different pressures. The volatile compounds and E-nose sensors were correlated with the PLS method analysis. When the pressure was at 400 MPa, the umami amino acids in the enzymatic hydrolysates of *S. rugoso-annulata* were 1.09 times higher, and the bitter amino acids were 7.65 times lower than those of the atmospheric pressure enzymatic hydrolysates. At 500 MPa, the sweet amino acids were 1.11 times higher than those of the atmospheric pressure enzymatic hydrolysates. At 400 MPa, 5′-GMP and 5′-IMP were 1.12 times and 1.15 times higher than those of atmospheric pressure enzymatic hydrolysates, which provided meat flavor and sweet aroma to the samples.

In conclusion, ultra-high pressure synergistic enzymatic hydrolysis can effectively improve the flavor of the enzymatic hydrolysates of *S. rugoso-annulata*, probably due to the changes in protein structure caused by UHP treatment, which exposed new enzymatic cleavage sites for the better binding of Flavourzyme^®^ to the substrate, thus promoting the release of flavor substances in *S. rugoso-annulata* and increasing the umami and sweetness. This thesis can provide a theoretical basis for the research of the flavor of *S. rugoso-annulata* and the development and utilization of *S. rugoso-annulata* products.

## Figures and Tables

**Table 1:** E-nose sensor performance description.

Array Serial Number	Sensor	Sensing Substance
1	W1C	Sensitive to aromatic compounds, benzene
2	W5S	High sensitivity to nitrogen oxides
3	W3C	Sensitive to ammonia and aromatic compounds
4	W6S	Selective for hydrogenation
5	W5C	Sensitive to short-chain alkanes, aromatic compounds
6	W1S	Sensitive to methyl groups
7	W1W	Sensitive to sulfides, pyrazines
8	W2S	Sensitive to alcohols, aldehydes and ketones
9	W2W	Sensitive to aromatic components, organic sulfides
10	W3S	Sensitive to long-chain alkanes

**Table 2:** Volatile composition in enzymatic hydrolysates of *S. rugoso-annulata* treated with different high pressure.

Count	RT	Volatile Compounds	RI	Content (μg/g)					
				Control	100 MPa	200 MPa	300 MPa	400 MPa	500 MPa
1	2.75	ethyl acetate	611.34	ND	0.0098 ± 0.0001 ^c^	0.0184 ± 0.0008 ^a^	0.0123 ± 0.0022 ^b^	0.0101 ± 0.0003 ^c^	ND
2	4.30	vinyl acetate	972.28	ND	0.0054 ± 0.0002 ^c^	0.0093 ± 0.0013 ^c^	0.0194 ± 0.0037 ^b^	0.0342 ± 0.0056 ^a^	0.0248 ± 0.0097 ^b^
3	15.69	hexyl formate	918.62	0.0069 ± 0.0012 ^b^	ND	ND	0.0022 ± 0.0008 ^c^	0.0090 ± 0.0010 ^a^	ND
4	22.38	γ-Valerolactone	958.24	0.0109 ± 0.0006 ^d^	0.0328 ± 0.0030 ^c^	0.0424 ± 0.0077 ^c^	0.0637 ± 0.0039 ^b^	0.0876 ± 0.0048 ^a^	0.0720 ± 0.0170 ^b^
5	22.85	gamma-Butyrolactone	915.25	0.0175 ± 0.0003 ^c^	0.0437 ± 0.0005 ^b^	0.0433 ± 0.0005 ^b^	0.0464 ± 0.0063 ^ab^	0.0536 ± 0.0062 ^a^	0.0535 ± 0.0038 ^a^
6	36.43	Dimethyl phthalate	1454.58	0.0069 ± 0.0020 ^e^	0.0234 ± 0.0011 ^d^	0.0953 ± 0.0046 ^a^	0.0798 ± 0.0044 ^b^	0.0282 ± 0.0030 ^d^	0.0372 ± 0.0074 ^c^
7	3.11	Valeraldehyde	715.01	0.0044 ± 0.0005 ^d^	0.0108 ± 0.0011 ^c^	0.0129 ± 0.0049 ^c^	0.0121 ± 0.0013 ^c^	0.0404 ± 0.0026 ^a^	0.0305 ± 0.0060 ^b^
8	7.19	Hexanal	1070.32	0.0331 ± 0.0043 ^d^	0.0387 ± 0.0008 ^cd^	0.0422 ± 0.0080 ^cd^	0.0493 ± 0.0028 ^bc^	0.0822 ± 0.0034 ^a^	0.0563 ± 0.0058 ^b^
9	12.56	3-Butanolal	895.21	0.0035 ± 0.0005 ^a^	ND	ND	ND	ND	ND
10	16.73	Nonanal	1104.74	0.0028 ± 0.0005 ^bc^	0.0087 ± 0.0008 ^abc^	ND	0.0105 ± 0.0092 ^ab^	0.0129 ± 0.0069 ^a^	ND
11	3.40	Ethanol	545.36	ND	0.1109 ± 0.0015 ^b^	0.1509 ± 0.0112 ^ab^	0.1279 ± 0.0725 ^b^	0.1946 ± 0.0158 ^b^	0.2127 ± 0.0341 ^a^
12	7.68	1-Octanol,2-butyl-	452.01	ND	ND	ND	ND	0.0159 ± 0.0075 ^a^	ND
13	13.72	Isopropanol	934.65	0.0030 ± 0.0002 ^b^	ND	0.0130 ± 0.0068 ^a^	ND	ND	ND
14	19.39	Ethylhexanol	1030.87	0.0117 ± 0.0004 ^b^	0.0153 ± 0.0025 ^b^	0.0270 ± 0.0080 ^a^	0.0292 ± 0.0091 ^a^	0.0362 ± 0.0050 ^a^	ND
15	20.74	2,3-Butanediol	789.46	0.0102 ± 0.0014 ^d^	0.0757 ± 0.0011 ^c^	0.1218 ± 0.0231 ^c^	0.1888 ± 0.0324 ^b^	0.2035 ± 0.0057 ^ab^	0.2264 ± 0.0266 ^a^
16	20.86	Linalool	1099.77	0.0092 ± 0.0002 ^b^	0.0132 ± 0.0013 ^b^	0.0245 ± 0.1535 ^a^	0.0344 ± 0.0058 ^b^	0.0406 ± 0.0028 ^b^	0.0516 ± 0.0104 ^b^
17	21.21	2-Heptanol	900.26	ND	ND	0.0087 ± 0.0012 ^a^	ND	0.0090 ± 0.0030 ^a^	ND
18	22.04	Propanediol	740.33	ND	ND	0.0447 ± 0.0077 ^b^	0.0452 ± 0.0054 ^b^	0.0587 ± 0.0034 ^ab^	0.0730 ± 0.0250 ^a^
19	28.54	Benzyl alcohol	1036.78	0.0057 ± 0.0008 ^b^	ND	ND	ND	0.0309 ± 0.0011 ^a^	ND
20	29.30	Phenethyl alcohol	1116.46	0.0070 ± 0.0008 ^d^	0.0257 ± 0.0025 ^c^	0.0238 ± 0.0040 ^c^	0.0497 ± 0.0095 ^b^	0.0633 ± 0.0079 ^a^	0.0664 ± 0.0066 ^a^
21	11.67	Oxalic acid	1023.01	ND	ND	0.0024 ± 0.0021 ^a^	ND	ND	ND
22	18.70	acetic acid	612.58	0.0241 ± 0.0003 ^d^	0.0287 ± 0.0016 ^d^	0.1841 ± 0.0110 ^c^	0.2354 ± 0.0369 ^bc^	0.2570 ± 0.0440 ^b^	0.3410 ± 0.0440 ^a^
23	21.60	Isobutyric acid	774.38	0.0087 ± 0.007 ^c^	0.0148 ± 0.0004 ^c^	0.1002 ± 0.0118 ^b^	0.1048 ± 0.0004 ^b^	0.1617 ± 0.0400 ^a^	0.1624 ± 0.0388 ^a^
24	23.98	Isovaleric acid	863.34	0.0176 ± 0.0007 ^d^	0.1527 ± 0.0005 ^bc^	0.1339 ± 0.0012 ^c^	0.1757 ± 0.0063 ^b^	0.2114 ± 0.0231 ^a^	0.2401 ± 0.0400 ^a^
25	27.89	Hexanoic acid	990.12	0.0049 ± 0.0020 ^b^	0.0087 ± 0.0008 ^b^	0.0165 ± 0.0013 ^ab^	0.0184 ± 0.0018 ^ab^	0.0262 ± 0.0026 ^a^	0.0237 ± 0.0184 ^a^
26	10.35	Pyridine	819.76	0.1750 ± 0.0060 ^cd^	0.1384 ± 0.0011 ^d^	0.2939 ± 0.0073 ^ab^	0.2580 ± 0.1100 ^abc^	0.3235 ± 0.0231 ^a^	0.2127 ± 0.0332 ^bcd^
27	13.52	Isohexadecane	326.15	ND	ND	ND	ND	0.0875 ± 0.0037 ^a^	ND
28	14.61	2,5-Dimethylpyrazine	923.12	0.0034 ± 0.0005 ^a^	0.0030 ± 0.0020 ^a^	0.0028 ± 0.0013 ^a^	ND	0.0018 ± 0.0003 ^ab^	ND
29	14.81	2,6-Dimethylpyrazine	917.03	0.0065 ± 0.0023 ^d^	0.0102 ± 0.0023 ^d^	0.0143 ± 0.0016 ^d^	0.0236 ± 0.0038 ^c^	0.0464 ± 0.0063 ^a^	0.0338 ± 0.0089 ^b^
30	17.69	cyclohexenone	920.51	ND	0.0012 ± 0.0012 ^b^	0.0121 ± 0.0013 ^a^	ND	ND	ND
31	23.02	N-Ethylacetamide	877.47	ND	0.0483 ± 0.0013 ^c^	0.0639 ± 0.0050 ^b^	0.0629 ± 0.0083 ^b^	0.0720 ± 0.0090 ^ab^	0.0786 ± 0.0026 ^a^
32	23.38	Ethylformamide	947.51	0.0028 ± 0.007 ^b^	ND	ND	ND	0.0145 ± 0.0126 ^a^	ND
33	24.39	2 H-Pyran-2-one,5,6-dihydro-	916.41	0.0287 ± 0.0008 ^e^	0.0931 ± 0.0034 ^d^	0.1089 ± 0.0087 ^d^	0.1741 ± 0.0091 ^c^	0.2738 ± 0.0263 ^a^	0.2417 ± 0.0289 ^b^
34	25.79	2(5 H)-Furanone	918.52	0.0140 ± 0.0019 ^e^	0.0321 ± 0.0028 ^d^	0.0476 ± 0.0074 ^c^	0.0583 ± 0.0038 ^b^	0.0752 ± 0.0041 ^a^	0.0681 ± 0.0061 ^a^
35	30.65	2-Acetyl pyrrole	1064.65	0.0151 ± 0.0002 ^a^	0.0201 ± 0.0008 ^a^	0.0219 ± 0.0022 ^a^	0.0250 ± 0.0170 ^a^	0.0289 ± 0.0058 ^a^	0.0245 ± 0.0030 ^a^
36	31.44	Phenol	980.38	0.0021 ± 0.0003 ^b^	ND	ND	ND	0.0059 ± 0.0064 ^a^	ND
37	31.87	2-Pyrrolidone	1076.53	0.0121 ± 0.0009 ^d^	0.0548 ± 0.0033 ^c^	0.0533 ± 0.0035 ^c^	0.0599 ± 0.0011 ^c^	0.1061 ± 0.002 ^a^	0.0905 ± 0.0097 ^b^
38	34.65	5-Isopropyl-2-methylphenol	1299.34	0.0032 ± 0.0003 ^b^	0.0022 ± 0.0010 ^bc^	0.0068 ± 0.0019 ^a^	0.0017 ± 0.0007 ^bc^	0.0011 ± 0.0018 ^bc^	ND

Note: a–e Different superscript letters in the same row imply significant differences between treatments. Each value is expressed as mean ± SD (n = 3). RT: retention time. RI: retention index. ND means no detectable.

**Table 3:** E-tongue sensor response values of enzymatic hydrolysates of *S. rugoso-annulata* treated with different ultra high pressure.

Sensors	Taste Value					
	Control	100 MPa	200 MPa	300 MPa	400 MPa	500 MPa
Bitterness	4.090 ± 0.030 ^d^	4.737 ± 0.078 ^b^	5.140 ± 0.133 ^a^	4.577 ± 0.091 ^c^	3.613 ± 0.101 ^e^	4.197 ± 0.004 ^d^
Aftertaste-B	−0.190 ± 0.050 ^b^	−0.167 ± 0.018 ^b^	−0.330 ± 0.011 ^c^	−0.197 ± 0.013 ^b^	−0.577 ± 0.079 ^d^	0.023 ± 0.011 ^a^
Umami	11.007 ± 0.343 ^d^	13.120 ± 0.015 ^bc^	13.267 ± 0.035 ^bc^	13.283 ± 0.119 ^b^	13.930 ± 0.051 ^a^	12.987 ± 0.016 ^c^
Richness	1.740 ± 0.110 ^e^	2.360 ± 0.061 ^b^	2.600 ± 0.016 ^a^	2.240 ± 0.080 ^c^	1.940 ± 0.041 ^d^	2.207 ± 0.006 ^c^
Saltiness	6.570 ± 0.080 ^a^	6.030 ± 0.036 ^b^	5.940 ± 0.004 ^b^	5.120 ± 0.118 ^c^	4.210 ± 0.020 ^e^	4.550 ± 0.095 ^d^
Sweetness	8.790 ± 0.070 ^b^	9.120 ± 0.022 ^a^	9.200 ± 0.253 ^a^	9.210 ± 0.205 ^a^	9.350 ± 0.052 ^a^	9.320 ± 0.085 ^a^

Note: a–e Different superscript letters in the same row imply significant differences between treatments. The significant differences are in rows. Each value is expressed as mean ± SD (n = 3).

**Table 4:** Content of free amino acids in enzymatic hydrolysates of *S. rugoso-annulata* treated with different ultra high pressure.

Amino Acid	Content (g/100 g)					
	Control	100 MPa	200 MPa	300 MPa	400 MPa	500 MPa
Aspartic acid (Asp)	2.54 ± 0.18 ^a^	2.53 ± 0.05 ^a^	2.54 ± 0.09 ^a^	2.55 ± 0.08 ^a^	2.59 ± 0.03 ^a^	2.59 ± 0.05 ^a^
Glutamic acid (Glu)	5.21 ± 0.23 ^b^	5.36 ± 0.10 ^ab^	5.44 ± 0.58 ^ab^	5.49 ± 0.04 ^ab^	5.89 ± 0.28 ^a^	5.61 ± 0.12 ^ab^
Threonine (Thr)	1.48 ± 0.10 ^a^	1.51 ± 0.06 ^a^	1.50 ± 0.20 ^a^	1.52 ± 0.12 ^a^	1.53 ± 0.09 ^a^	1.52 ± 0.02 ^a^
Serine (Ser)	1.52 ± 0.06 ^a^	1.58 ± 0.06 ^a^	1.62 ± 0.06 ^a^	1.65 ± 0.07 ^a^	1.67 ± 0.04 ^a^	1.62 ± 0.16 ^a^
Glycine (Gly)	1.48 ± 0.09 ^a^	1.52 ± 0.10 ^a^	1.54 ± 0.06 ^a^	1.48 ± 0.05 ^a^	1.49 ± 0.03 ^a^	1.51 ± 0.13 ^a^
Proline (Pro)	1.20 ± 0.16 ^b^	1.19 ± 0.17 ^b^	1.21 ± 0.12 ^b^	1.23 ± 0.11 ^b^	1.24 ± 0.05 ^b^	1.56 ± 0.06 ^a^
Alanine (Ala)	2.54 ± 0.08 ^c^	2.72 ± 0.13 ^ab^	2.77 ± 0.21 ^ab^	2.78 ± 0.24 ^ab^	2.91 ± 0.11 ^a^	2.93 ± 0.06 ^a^
Phenylalanine (Phe)	1.45 ± 0.08 ^a^	1.39 ± 0.03 ^ab^	1.34 ± 0.06 ^abc^	1.28 ± 0.04 ^bc^	1.23 ± 0.08 ^c^	1.26 ± 0.06 ^c^
Histidine (His)	1.35 ± 0.14 ^a^	1.29 ± 0.01 ^a^	1.25 ± 0.06 ^a^	0.98 ± 0.14 ^b^	0.97 ± 0.05 ^b^	1.03 ± 0.09 ^b^
Valine (Val)	1.85 ± 0.07 ^a^	1.82 ± 0.14 ^ab^	1.75 ± 0.06 ^abc^	1.62 ± 0.03 ^c^	1.60 ± 0.11 ^c^	1.66 ± 0.12 ^bc^
Methionine (Met)	0.38 ± 0.14 ^a^	0.38 ± 0.07 ^a^	0.36 ± 0.09 ^a^	0.29 ± 0.07 ^a^	0.25 ± 0.09 ^a^	0.24 ± 0.08 ^a^
Isoleucine (Ile)	1.61 ± 0.02 ^a^	1.59 ± 0.11 ^a^	1.35 ± 0.06 ^bc^	1.42 ± 0.03 ^b^	1.25 ± 0.09 ^c^	1.27 ± 0.08 ^c^
Leucine (Leu)	2.49 ± 0.12 ^a^	2.22 ± 0.19 ^ab^	2.09 ± 0.03 ^b^	1.98 ± 0.22 ^b^	1.99 ± 0.13 ^b^	2.04 ± 0.17 ^b^
Arginine (Arg)	1.02 ± 0.01 ^a^	0.84 ± 0.12 ^a^	0.39 ± 0.12 ^b^	0.42 ± 0.11 ^b^	0.36 ± 0.09 ^b^	0.30 ± 0.20 ^b^
Tyrosine (Tyr)	0.98 ± 0.11 ^a^	0.99 ± 0.23 ^a^	1.11 ± 0.10 ^a^	1.08 ± 0.76 ^a^	1.00 ± 0.22 ^a^	1.00 ± 0.02 ^a^
Lysine (Lys)	1.29 ± 0.10 ^c^	1.32 ± 0.02 ^bc^	1.52 ± 0.04 ^a^	1.48 ± 1.04 ^ab^	1.31 ± 0.01 ^bc^	1.41 ± 0.15 ^abc^
Cysteine (Cys)	1.21 ± 0.15 ^bc^	1.34 ± 0.06 ^ab^	1.47 ± 0.06 ^a^	1.02 ± 0.72 ^cd^	0.96 ± 0.15 ^d^	1.06 ± 0.06 ^cd^
Umami	7.75 ± 0.09 ^c^	7.89 ± 0.11 ^c^	7.98 ± 0.05 ^b^	8.04 ± 0.06 ^b^	8.48 ± 0.25 ^a^	8.20 ± 0.07 ^ab^
Sweet	8.22 ± 0.12 ^d^	8.52 ± 0.02 ^d^	8.64 ± 0.02 ^c^	8.66 ± 0.15 ^c^	8.84 ± 0.16 ^b^	9.14 ± 0.12 ^a^
Bitter	10.15 ± 0.16 ^a^	9.53 ± 0.07 ^ab^	8.53 ± 0.03 ^b^	7.99 ± 0.34 ^b^	7.65 ± 0.05 ^c^	7.8 ± 0.09 ^bc^

Note: a–d Different superscript letters in the same row imply significant differences between treatments. Each value is expressed as mean ± SD (n = 3). Umami: calculated from the sum of ASP and Glu. Sweet: calculated from the sum of Thr, Ser, Gly, Pro and Ala. Bitter: calculated from the sum of Phe, His, Val, Met, Ile, Leu, and Arg.

**Table 5:** Content of 5′-nucleotides in enzymatic hydrolysates of *S. rugoso-annulata* treated with different ultra high pressure.

5′-Nucleotides	Content (mg/100 g)					
	Control	100 MPa	200 MPa	300 MPa	400 MPa	500 MPa
CMP	411.753 ± 0.251 ^c^	412.024 ± 0.288 ^c^	395.293 ± 0.331 ^d^	413.162 ± 0.398 ^b^	414.409 ± 0.594 ^a^	412.711 ± 0.147 ^b^
GMP	4.785 ± 0.126 ^b^	4.896 ± 0.327 ^b^	4.823 ± 0.279 ^b^	5.027 ± 0.041 ^ab^	5.364 ± 0.162 ^a^	5.032 ± 0.181 ^ab^
IMP	37.497 ± 0.495 ^f^	39.243 ± 0.757 ^d^	40.075 ± 0.206 ^c^	40.891 ± 0.132 ^b^	43.098 ± 0.027 ^a^	38.298 ± 0.123 ^e^
UMP	23.419 ± 0.477 ^b^	23.512 ± 0.015 ^a^	23.579 ± 0.149 ^a^	23.075 ± 0.046 ^bc^	21.914 ± 0.096 ^c^	22.379 ± 0.516 ^b^

Note: a–f Different superscript letters in the same row imply significant differences between treatments. Each value is expressed as mean ± SD (n = 3). CMP: 5′-cytosine monophosphate; UMP: 5′-uridine monophosphate; GMP: 5′-guanosine monophosphate; IMP: 5′-inosine monophosphate.

## Data Availability

Data is contained within the article.
